# Advancements in Brain Research: The In Vivo/In Vitro Electrochemical Detection of Neurochemicals

**DOI:** 10.3390/bios14030125

**Published:** 2024-02-26

**Authors:** Xiaoxuan Xu, Yimei Zuo, Shu Chen, Amir Hatami, Hui Gu

**Affiliations:** 1 Key Laboratory of Theoretical Organic Chemistry and Functional Molecule of Ministry of Education, School of Chemistry and Chemical Engineering, Hunan University of Science and Technology, Xiangtan 411201, China; yummy0212@yeah.net (X.X.); zuoyimei1999@163.com (Y.Z.); hgu@hnust.edu.cn (H.G.); 2 Department of Chemistry, Institute for Advanced Studies in Basic Sciences (IASBS), Prof. Sobouti Boulevard, P.O. Box 45195-1159, Zanjan 45137-66731, Iran; 3 Department of Chemistry and Molecular Biology, University of Gothenburg, 405 30 Gothenburg, Sweden

**Keywords:** neurochemicals, bioanalysis, microelectrode

## Abstract

Neurochemicals, crucial for nervous system function, influence vital bodily processes and their fluctuations are linked to neurodegenerative diseases and mental health conditions. Monitoring these compounds is pivotal, yet the intricate nature of the central nervous system poses challenges. Researchers have devised methods, notably electrochemical sensing with micro-nanoscale electrodes, offering high-resolution monitoring despite low concentrations and rapid changes. Implantable sensors enable precise detection in brain tissues with minimal damage, while microdialysis-coupled platforms allow in vivo sampling and subsequent in vitro analysis, addressing the selectivity issues seen in other methods. While lacking temporal resolution, techniques like HPLC and CE complement electrochemical sensing’s selectivity, particularly for structurally similar neurochemicals. This review covers essential neurochemicals and explores miniaturized electrochemical sensors for brain analysis, emphasizing microdialysis integration. It discusses the pros and cons of these techniques, forecasting electrochemical sensing’s future in neuroscience research. Overall, this comprehensive review outlines the evolution, strengths, and potential applications of electrochemical sensing in the study of neurochemicals, offering insights into future advancements in the field.

## 1. Introduction

Brain neurochemicals constitute a range of crucial chemicals for the central nervous system’s function, collectively encoding brain activities in physiological and pathological processes [1,2]. These neurochemicals intricately regulate the nervous system, ensuring its proper function and significantly contributing to signaling, learning, motor control, and even treating neurological diseases. The evolution of advanced in vitro neurochemical detection technologies has revolutionized our comprehension of neurochemical action mechanisms. However, these assays pose challenges, like potential neurochemical degradation and limitations in understanding real-time dynamic bioprocesses. Consequently, quantitatively monitoring brain neurochemicals in vivo holds profound significance in understanding human cognitive brain function.

The complexity of the brain’s environment and the fluctuating nature of neurochemicals during different physiological and pathological processes set stringent requirements for analytical methods in in vivo cerebral neurochemical monitoring. Within this domain, two major categories—implanted microsensors and microdialysis sampling—have demonstrated robust application prospects [3]. These technologies have rapidly advanced in recent years, fortifying in vivo neurochemical analysis. The popularity of microelectrodes in neuroscience surged after Gilbert Ning Ling successfully developed glass microelectrodes with apertures smaller than 1 micron. These microelectrodes, due to their minute size, allow brain implantation with reduced damage while providing excellent temporal and spatial resolutions [4,5]. In the late 1960s, Ralph Adams [6] and colleagues studied the electrochemical behavior of various biogenic amines and implanted a carbon paste electrode in an anesthetized rat’s brain, marking the initial attempt to monitor neurochemicals using conventional voltammetric techniques. Despite initially recording electrical signals likely from ascorbic acid instead of dopamine, this work crucially demonstrated that neurochemicals could diffuse onto electrode surfaces, inspiring researchers to explore microelectrodes for in vivo neurochemical measurements. Then, fast scan cyclic voltammetry (FSCV), introduced by Wightman in 1981, offered groundbreaking insights into neurochemical analysis, enabling ultra-high temporal and spatial resolution on-site signal output [7].

Moreover, various electrochemical techniques, including differential pulse voltammetry, amperometry, among others, have been employed for the in vivo monitoring of chemical neuro-substances [8,9,10,11,12,13], laying a solid foundation for microelectrode applications in neurochemistry. Initially focused on electroactive molecules, like ascorbic acid and 5-hydroxytryptamine, microelectrode-based research encountered challenges with substances like catecholamines due to an overlapping electrochemical redox potential. However, recent advancements in microelectrodes designed to recognize molecules via enzymes, aptamers, or electrochemical probes have significantly overcome these hurdles, and these electrodes have been used for monitoring non-electroactive molecules. For example, Gerhard’s team, in 2001, successfully monitored non-electrochemically active glutamate molecules in the rat prefrontal cortex using glutamate oxidase through a self-referencing microelectrode array (MEA) platform [14].

Despite many advantages, microelectrodes are susceptible to the biological contamination of the tissue microenvironment and struggle with quantitatively determining neurochemicals at basal levels [15,16]. Nonetheless, these limitations have not notably diminished the prominence of microsensors compared to other analytical techniques [17,18,19,20,21,22].

In parallel, microdialysis, introduced in the late 1950s, transformed our understanding of in vivo neurochemicals by measuring endogenous compound concentrations in animal brains [17,18]. This technology, often combined with instruments like high-performance liquid chromatography (HPLC) or capillary electrophoresis (CE), enhances selectivity and time resolution. Continuous efforts in coupling analytical methods with microdialysis have substantially improved its time resolution to seconds, challenging the initial notion of a poor time resolution associated with microdialysis [22,23,24,25]. Recent advancements, especially coupling microdialysis with biosensors [26,27,28], have revolutionized its capabilities. This combination leads to a sensitive analysis, low detection limit, and prevents analyte degradation. For example, in 2001, M. M. Rhemrev-Boom’s group utilized mobile biosensors for direct-coupled continuous low-flow microdialysis, showcasing enhanced analyte selectivity for glucose and lactic acid [29].

In summary, the review aims to analyze the suitability and advantages of implanted microsensors and microdialysis for the in vivo analysis of neurochemicals in brain samples. This review offers readers a comprehensive understanding to effectively choose analytical methods, fostering advancements in neurotransmitter analysis that benefit downstream research, disease diagnosis, drug discovery, neurochemicals, associated diseases, and treatment.

## 2. Types of Neurochemicals and Associated Diseases

**Figure 1:**
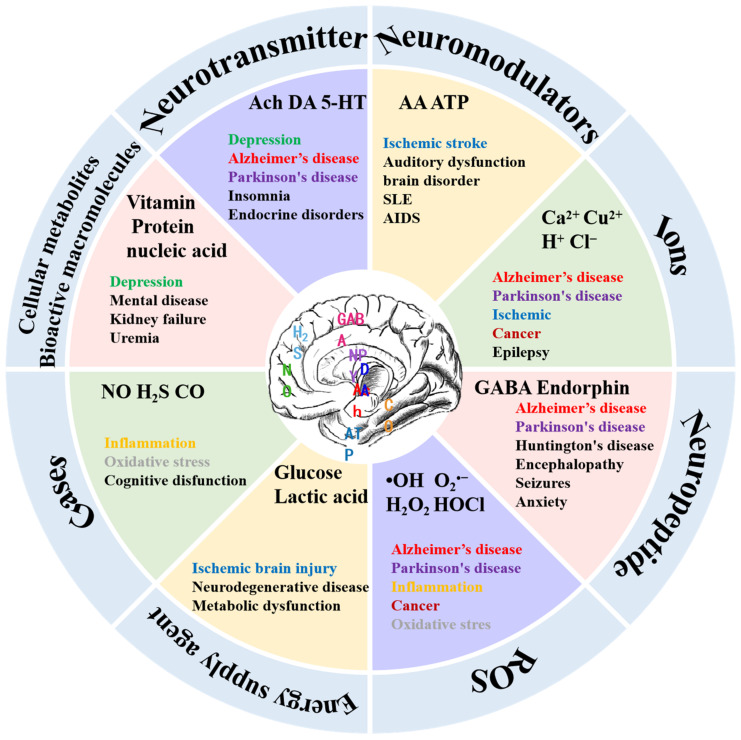
Classification of neurochemicals and associated diseases. The figure contains only some typical neurochemicals, not all of them (Ach: acetylcholine; DA: dopamin; 5-HT: 5-hydroxytryptamine; AA: ascorbic acid; ATP: adenosine triphosphate; SLE: systemic lupus erythematosus; AIDS: acquired immune deficiency syndrome; GABA: γ-aminobutyric acid; •OH: hydroxyl radical; H_2_O_2_: hydrogen peroxide; O_2_^•−^ superoxide radical; HOCl: hypochlorous acid; NO: nitric oxide; H_2_S: hydrogen sulfide; CO: carbon monoxide.).

The brain, composed of billions of neurons and neuroglia cells, hosts a diverse array of neurochemicals that continuously interact, forming a dynamic neural network responsible for regulating consciousness and behavior. These neurochemicals fall into two main classifications: neurotransmitters and neuromodulators. Neurotransmitters act as messengers, facilitating information transfer between synapses through direct electrical contact and converting action potentials into chemical signals. In contrast, neuromodulators often regulate neurotransmission and some bioprocesses. Neurochemicals encompass small ions, gases, reactive oxygen species (ROS), energy suppliers, peptides, and bioactive macromolecules, collectively governing vesicles, neurons, and circuits. They play specific roles in regulating daily physiological behavior, and imbalances in neurochemicals can lead to diseases, disrupting organism functions (Figure 1).

Neurotransmitters, like dopamine (DA), serotonin (5-HT), epinephrine (E), norepinephrine (NE), glutamate, and acetylcholine, amplify, transmit, and convert signals within cells, influencing mood regulation, cognition, memory formation, learning, and motor control. Catecholamine neurotransmitters, including DA and E, are extensively investigated, with alterations in their concentrations linked to neurodegenerative and psychiatric disorders [30,31]. Oscillating NE concentrations correlate with disorders like Parkinson’s disease and attention deficit hyperactivity disorder [32]. Reduced levels of 5-hydroxytryptophan are associated with depression, insomnia, and endocrine disruptions [33,34]. A decline in acetylcholine is linked to age-related memory loss and Alzheimer’s disease. Glutamate (Glu) contributes to neuronal excitation, with excessive levels leading to excitotoxicity and damaging and killing nerve cells [35]. Gamma-aminobutyric acid (GABA), a primary inhibitory neurochemical, plays a pivotal role in overall neuronal function. Fluctuations in Glu and GABA levels serve as markers for various neurological and psychiatric disorders [36,37,38].

In what follows, we review neuromodulators and their roles in our body. Ascorbic acid (AA) and extracellular adenosine triphosphate (ATP) are recognized neuromodulators and play critical roles in physiological and pathological processes. The antioxidant properties of AA scavenge reactive oxygen species, reducing oxidative stress and exerting neuroprotective effects. Variations in AA levels indicate neurodegenerative diseases, and its deficiency may lead to limb weakness, depression, bone pain, and osteoporosis. Dysregulated AA concentrations trigger diseases like ischemic stroke, auditory dysfunction, and olfactory dysfunction [39,40,41,42]. ATP, the universal energy currency, regulates physiological processes through purinergic receptor activation. Abnormal ATP levels are linked to immune function impairment in diseases like rheumatoid arthritis and AIDS.

Various ions (Ca^2+^, Cu^2+^, Na^+^, H^+^, and Cl^−^) play vital roles as neuromodulators in cellular signaling, molecular structure formation, and (co)enzyme activation. Metal ions chelate with biomolecules, enhancing activity and stability or act as redox centers, impacting biological processes. Dysregulated ion levels correlate with neurodegenerative diseases, cancer, and diabetes. Anions like chloride and bicarbonate regulate cell volume, membrane potential, and vesicle pH, with alterations linked to diseases such as cystic fibrosis and myasthenia gravis [43,44,45,46]. Among them, H^+^ ion balance is crucial for monitoring the body’s acid–base balance, with deviations triggering conditions like epilepsy, ischemia, and psychiatric disorders [47,48,49].

Neurochemicals encompass soluble gases, like nitric oxide (NO), hydrogen sulfide (H_2_S), and carbon monoxide (CO), known as gas transmitters. These lipophilic, soluble molecules readily cross cell membranes and are synthesized only as needed. Gas transmitters impact neuropsychiatric disorders, like anxiety. For example, nitric oxide enhances long-term synaptic transmission, influencing learning and memory. Hydrogen sulfide (H_2_S) regulates gamma-aminobutyric acid B receptor receptors, pH balance, and calcium homeostasis and provides neuroprotection against oxidative stress [50,51,52,53,54]. Abnormal concentrations of CO are associated with inflammation, liver disease, diabetes, and cancer [55,56,57,58,59].

Reactive oxygen species (ROS), generated from oxygen-containing molecules, play crucial roles in physiological and pathological processes. ROS accumulation causes oxidative stress, inflammation, and cellular damage, leading to necrosis and cancer.

Glucose and lactic acid serve as primary energy sources for the brain. A dysfunctional glucose metabolism correlates with neuropathologies like ischemic brain injury and neurodegenerative disorders. Lactate contributes to brain energy metabolism, regulating microcirculation and neuronal excitability and offering neuroprotection [60,61].

Another group of neuromodulators is neuropeptides, categorized as hypothalamic and pituitary, impacting synaptic transmission, neuronal inhibition, cognitive impairment, and stress response [62,63,64]. For example, the neuropeptide γ is associated with inhibiting the transmission of excitatory amino acids and reducing neuronal excitation. Endorphins affect anxiety and pain perception.

The last group of neuromodulators comprises metabolites. Generally, cell metabolism produces metabolites, like lipids, vitamins, antibiotics, toxins, and hormones, impacting neuromodulation. An abnormal brain metabolism often coexists with psychiatric disorders, like major depressive disorder (MDD) [65].

**Figure 2:**
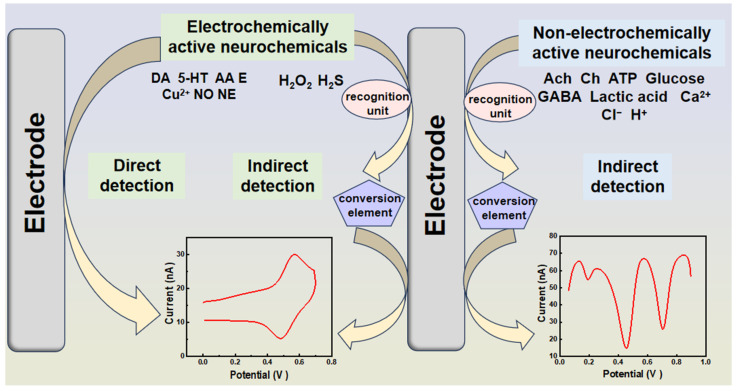
Electrochemical sensing strategies for the direct and indirect detection of neurochemicals.

As previously mentioned, neurochemical imbalances can cause extensive harm to organisms. Analyzing their concentrations and monitoring their interactions under pathophysiological conditions at the molecular level aids in understanding brain function, guiding diagnosis, and the treatment of neurological diseases. Among all analytical techniques, electrochemical analytical methods stand out due to their speed, sensitivity, cost effectiveness, and ability to enable online detection. These methods rely on the direct redox of electrochemically active analytes on electrode surfaces or specific recognition units for indirect detection. However, the complex in vivo environment often requires an indirect detection strategy for some electrically active molecules to prevent sensor toxicity (Figure 2).

## 3. In Vivo Electrochemical Measurements of Neurochemicals in the Brain Tissue

**Figure 3:**
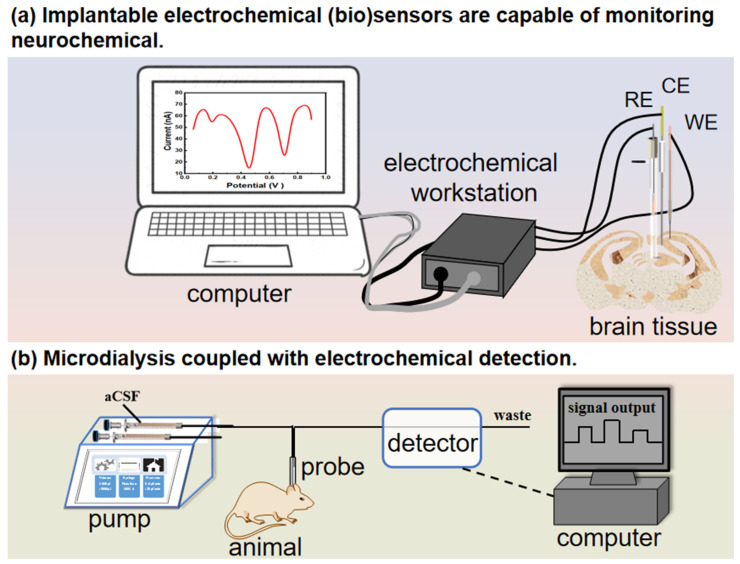
Two analytical methods for the in vivo electrochemical detection of dynamic changes in neurochemicals. (**a**) Implantation of electrochemical biosensors in brain regions to monitor real-time dynamic changes in neurochemical levels. (**b**) In vivo sampling for offline or online detection using microdialysis coupled with an electrochemical system.

Two main types of analytical methods are commonly utilized to electrochemically detect dynamic changes in neurochemicals in vivo (Figure 3). The first method involves implantable in vivo electrochemical (bio)sensing, where miniature electrochemical sensors are directly implanted into brain regions to record real-time dynamic changes in neurochemical levels within the central nervous system (CNS). The second method involves in vivo sampling-based neurochemical analysis, typically encompassing the electroanalysis of neurochemicals sampled in vivo from brain regions in vitro. This process relies on in vivo microdialysis combined with electrochemical measurements [25,66,67], alongside sample separation and offline assays. These techniques allow for multiple neurochemical analyses to be conducted simultaneously. Alternatively, selective online assays enable the continuous monitoring of one or more neurochemicals. In this section, we discuss the research progress in detecting neurochemicals in the brain tissue using two primary techniques: implantable microelectrodes and microdialysis (Figure 3).

### 3.1. Implantable Electrochemical Biosensors to Monitor Neurochemicals in the Brain Tissue

Implantable electrochemical biosensors are considered highly promising and effective technologies for real-time analyte monitoring due to their excellent spatial and temporal resolution as well as adaptable electrode interfaces. The commonly used electrode materials are divided into carbon and metal. Due to the complexity of the in vivo environment, carbon-based materials are widely used for the detection of neurochemicals in vivo because of their inertness. Common carbon electrodes include glass carbon electrodes (GCEs), carbon paste electrodes (CPEs), and screen-printed carbon electrodes. However, the most popular electrodes for neurochemical measurements are carbon fiber microelectrodes (CFMEs) because of their excellent biocompatibility, small size, and good electron transfer of neurotransmitters. In order to reduce tissue damage and inflammatory reactions during implantation, the size of the electrodes is crucial and can be regulated down to the micrometer and even nanometer levels [68,69]. Generally, electrode geometries are limited to discs, cylinders, or cones. Advances in nanolithography and 3D printing allow electrodes to have customizable geometries as well as optimized chemical and surface structures, which greatly improve the performance of the electrodes. Additionally, the advancement in microelectrode arrays facilitates the placement of multiple micrometer-sized electrodes on the same device, enabling the simultaneous detection of specific neurochemical molecules across various brain regions.

Electrochemically active substances can undergo direct redox reactions at the electrode interface, generating electrical signals for direct monitoring. However, non-electrochemically active neurochemicals require specific recognition units to capture and then convert chemical signals into electrical signals for indirect detection, because the redox potentials of some electrically active neurochemicals overlap extensively in vivo, and indirect monitoring methods are also available to improve selectivity. The in vivo neural environment is complex, featuring low analyte concentrations and thousands of potential interfering compounds, making sensitivity and selectivity crucial challenges in designing effective electrochemical sensors. In this way, advancements in various nanomaterials and recognition units have been instrumental in enhancing sensitivity and selectivity for detecting various neurochemicals.

#### 3.1.1. Non-Electroactive Neurochemicals

The detection of neurochemicals typically involves directly measuring the corresponding current generated by the target species on the electrode surface, providing a quantitative assessment of dynamic chemical changes. Electrochemically active neurochemicals can be reoxidized directly on the electrode surface. However, the detection of non-electroactive neurochemicals (e.g., choline, acetylcholine, and glutamate) necessitates modified sensors. These sensors require a recognition unit capable of interacting with the target and producing an electroactive molecule, such as H_2_O_2_, or transferring the signal to the electrochemical process, like an intermediate [70]. This capability is achieved through enzymes [71,72], aptamers, and electrochemical probes to complete the capture of the target analyte signal (refer to Table 1). Next, we present a summary of these strategies.

**● Enzymes** intrinsically possess catalytic activity and exhibit specific recognition toward analytes. Enzyme-based biosensors usually rely on a medium that facilitates electron transfer (MET), generating an electrical signal by oxidizing or reducing electrically active substances on the electrode surface. The resulting current is directly proportional to the analyte concentration, enabling the detection of the target analyte. Examples include choline oxidase and glutamate oxidase, known for their simplicity, stability, and high sensitivity. O. Frey’s group [86] used a semipermeable m-phenylenediamine layer to significantly enhance sensor immunity to interference. They developed a micro-biosensor for the simultaneous detection of glutamate and choline in the rat brain using in silico process technology, opening new directions for detecting these substances within physiologically relevant concentration ranges. For reliable in vivo detection, Gerhard’s group [14] created a self-referencing microelectrode array (MEA) platform. By introducing a self-reference electrode and subtracting its current signal as background noise, interference from other species was eliminated. MEAs have been extensively used to measure rapid changes in glutamate levels in anesthetized and awake animals [14,87,88,89,90,91,92,93,94,95], lactate and glucose levels, and for the real-time monitoring of choline, acetylcholine (ACh) [96], and oxygen [97] in vivo.

**Figure 4:**
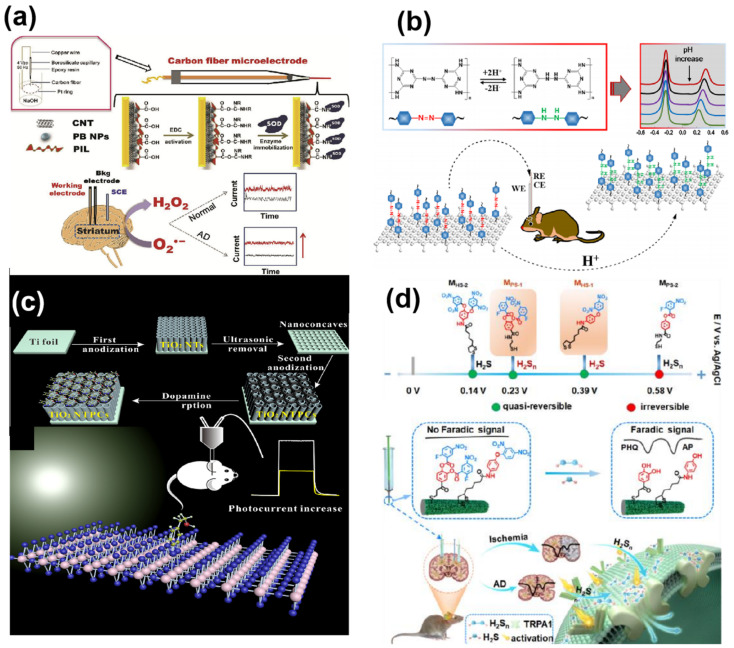
Implantable electrochemical microsensors for in vivo analysis. (**a**) Schematic diagram of the fabrication of an enzyme-based electrochemical microsensor for the determination of superoxide anion. (The black line represents the background current, which increases with the presence of superoxide radicals, as shown by the red line). Reprinted from Ref. [98] with permission. Copyright 2019 Elsevier B.V. (**b**) Diagram of an electrochemical microsensor strategy for rat brain pH measurement. (Different colored DPV curves represent tests at different pH, and the pH decreases from the top down). Reprinted from Ref. [76] with permission. Copyright 2021 American Chemical Society. (**c**) Molecularly imprinted polymer-based PEC-sensing platform for DA detection. Reprinted from Ref. [117] with permission. Copyright 2016 Elsevier B.V. (**d**) The working principle of the sensing strategy and specific probes for the simultaneous detection of H_2_S and H_2_Sn in the living mouse brain. Reprinted from Ref. [9] with permission. Copyright 1999–2023 John Wiley & Sons, Inc.

The ideal enzyme-based sensor capable of direct electron transfer (DET) from the redox-active center to the electrode surface belongs to the DET enzyme sensors category. DET enzyme sensors remain unaffected by changes in ambient oxygen concentration or additional mediators, making them adaptable to complex in vivo environments, ideal for neurochemical detection. Yu’s group [98] contributed significantly to ROS detection using functionalized ionic liquid polymers (PILs) coated on Prussian blue nanoparticles and carbon nanotubes (CNTs), enhancing sensor sites for SOD to improve sensitivity and stability to low O_2_^•−^ concentrations (Figure 4a). Hence, the sensor effectively tracked changes in O_2_^•−^ levels under normal and pathological conditions in the living brain system. Overall, enzymes play a crucial role in in vivo neurochemical detection owing to their unique selectivity and rapid kinetics. The development of various enzymes has expanded the scope of detectable analytes, and the emergence of synthetic nano-enzymes [99] holds promise for the future of biosensors.

**● Aptamers**, single-stranded DNA or RNA molecules from randomly sequenced nucleic acid pools, have gained attention for their high affinity and specificity to the target. Compared to enzymes, they offer convenient synthesis, design flexibility, and chemical stability. Aptamer-based electrochemical sensors undergo conformational changes when the aptamer on the electrode surface combines with the ligand, triggering electron transfer between the electrode and the modified REDOX group on the aptamer [100]. Currently, aptamers are used to detect neurochemicals, like DA [101], 5-HT [102], and ATP [103]. Aptamers possess a good biological compatibility. Due to a complex screening process, only specific neurochemicals have aptamer recognition units. In 2005, Yi Xiao et al. [104] pioneered an electrochemical aptamer sensor, sparking research interest. Subsequently, various aptamer electrochemical sensors have been developed, mostly for blood analyte detection [105,106]. However, few electrochemical aptamers have been applied successfully to brain tissue detection. Cui’s group [107] developed an electrochemical aptamer-based in vivo cocaine sensor on a silica-based neurorecording probe platform capable of measuring cocaine directly from discrete brain locations using square wave voltammetry, capturing real-time cocaine transient events in multiple brain regions over the entire pharmacokinetic time course.

Despite the many advantages of aptamers, several challenges persist, including limited measurement duration and obtaining high-performance aptamers for new targets. New aptamers need adaptation to sensing platforms, potentially bottlenecking sensor development for novel targets. Specificity remains a challenge; aptamers often bind chemical groups present in multiple targets, leading to cross-reactivity during detection [108,109]. This presents challenges for in vivo monitoring. The potential duration of in vivo aptamer-based measurements remains uncertain; prolonged in vivo measurements risk signal loss and electrode contamination, mitigated by surface coating or increasing single-layer packaging density to reduce protein contamination [110,111,112]. Despite being in its infancy, aptamer-based in vivo detection offers excellent time resolution, miniaturization, and other advantages, holding potential for in situ detection platforms.

**● Recognition Elements for Ions** play a crucial role in maintaining the central nervous system’s normal functioning. For instance, calcium ions act as second messengers in neurotransmitter regulation, while dysregulated Cl^−^ levels and brain pH disorders are associated with various neurological disorders. Efforts have been made to detect ions accurately in vivo. To enhance in vivo detection accuracy, a dual-channel recognition strategy using ratiometric microelectrodes was proposed for real-time monitoring. Fan Zhao et al. [113] achieved dual-channel recognition by modifying recognition elements on different microelectrodes, enabling the real-time in situ monitoring of pH in the rat brain, minimizing brain damage and inaccuracies between the electrodes. Gu’s group [76] constructed a ratiometric microsensor for pH monitoring, utilizing electrochemically oxidized graphene oxide (EOGO) to generate a built-in correction signal and a poly(melamine) (PMel) film as a pH-selective recognition membrane (Figure 4b). PMel, a highly pH-sensitive conductive polymer, facilitated the successful real-time monitoring of rat brain pH post-whole-brain ischemia/reperfusion events, confirming the robustness of the proposed ratiometric electrochemical microsensor platform.

**● Molecularly Imprinted Polymers (MIPs)**, formed by a template molecule’s size, shape, and functional groups, draw inspiration from the specific binding of antigens and antibodies—the ‘lock and key’ mechanism. These polymers, like a template, can absorb specific targets that fit the fabricated template in terms of shape, size, and chemical function. Due to their distinct structure and specific recognition ability or high selectivity, MIPs find extensive use, particularly in molecularly imprinted electrochemical sensors (MIPESs) [114]. Mosbach and Haupt [115] pioneered the integration of electrochemical sensors with MIPs, introducing MIPESs in 1999. Since then, MIPESs have detected organic compounds, heavy metal ions, emerging pollutants, and in vitro biomolecules. In 2012 [116], the first in vivo microsensor based on a MIP detected dopamine in the rat brain. Subsequently, focusing on neurochemical detection in the body, it has been employed for selective DA detection (Figure 4c) [117], as well as norepinephrine (NE) [118] and epinephrine (EP) [119].

**● Electrochemical Probes**: While in vitro environments can be corrected, the complexity of in vivo environments poses a risk to the selectivity of in vivo assays. To address this issue, electrochemical probes based on a dual recognition strategy were proposed for in vivo measurements, enhancing assay selectivity through the synergistic chemical recognition of specific ligands and the redox activity of chemicals. Recent studies suggest that biological activities initially attributed to hydrogen sulfide may actually be mediated by H_2_Sn, which converts endogenous hydrogen sulfide to hydrogen polysulfide in the presence of ROS. H_2_Sn is hypothesized to have a stronger oxidative capacity and reactivity than hydrogen sulfide, potentially being the true regulator in cellular signaling [120,121]. The further development of sensors that selectively detect multiple neurochemicals simultaneously is required for an in-depth understanding of in vivo molecular mechanisms. Despite the direct oxidation of H_2_S on the electrode surface, the slow oxidation of other electroactive biomolecules and the organism’s internal complexity cause significant interference. Tian’s group [9] designed two electrochemical probes, 3,4-bis((2-fluoro-5-nitrobenzoyl)oxy)-benzoic acid and N-(4-(2,5-dinitrophenoxy) phenyl)-5-(1, 2-dithiolan-3-yl)pentanamide, specifically recognizing H_2_Sn and hydrogen sulfide, respectively. Co-assembling these probes on a mesoporous gold membrane yielded a microsensor that responded well to both hydrogen sulfide (0.2–50 µM) and H_2_Sn (0.2 to 40 µM) (Figure 4d). This study contributed to understanding the molecular mechanisms of H_2_Sn and hydrogen sulfide and emphasized the significance of simultaneous detection for elucidating their pathophysiological roles in the brain.

**● Electrochemical Microarray Detection**, an electrochemical microarray detection platform, offers a promising approach to simultaneously sense multiple ions in the body under specific physiological or pathological conditions. Tian’s group [81] developed an electrochemical physiological microarray (ECPM) to quantify K^+^, Ca^2+^, and Na^+^ concentrations and pH using an open-circuit potentiostatic method. Different ion-recognizing elements designed for simultaneous electrical signal recording without cross-talk were employed. An internal reference electrode coated with a polyvinyl chloride membrane avoided the complex brain environment interference, ensuring the accuracy and selectivity of the developed ECPM. It provides new paths for the real-time monitoring of K^+^, Ca^2+^, and Na^+^ ions’ dynamics and quantitative concentrations, establishing the correlation between electrical and chemical signals.

#### 3.1.2. Electroactive Neurochemicals

Electrochemical sensing for detecting neurochemicals typically involves the direct oxidation or reduction of the target species on the electrode surface, generating corresponding electric currents that quantify dynamic chemical changes. Electrochemically active neurochemicals undergo direct oxidation or reduction on the electrode surface, producing detectable electrical signals. However, challenges arise due to the presence of multiple redox substances and the overlapping peak potentials of their reactions, hindering selective monitoring.

Nevertheless, functional brain regulatory networks enable electrochemical monitoring. Techniques, such as cyclic voltammetry (CV), chronoamperometry (CA), and potentiometric pulse (DPV), are commonly employed. CV is the recording of current–potential curves by controlling the electrode potential at different rates over time with one or more repeated scans in a triangular waveform. The basic principle is that a pulsed voltage with a triangular waveform is applied to the closed loop formed by the working electrode and the counter electrode to change the potential at the working electrode/electrolyte interface at a certain rate, forcing the active material at the working electrode to undergo an oxidation/reduction reaction. CA monitors the gain or loss of electrons in the presence of a fixed potential. In addition to a three-electrode setup consisting of a working electrode, a reference electrode, and a counter electrode, it can also be performed using a two-electrode setup with the reference and counter short-circuited together. The differential current of DPV is a symmetrical volt–ampere peak whose intensity is proportional to the concentration of the analyte. Due to the high Faraday to charging current ratio of the DPV, the direct detection of the base analyte at low concentrations can be achieved. Fast scan cyclic voltammetry (FSCV) stands out due to its scanning potentials and high scan rate, enabling the detection of small changes in neurochemical concentrations and differentiation based on unique redox potentials. In their study, Butcher et al. [122] utilized carbon fiber microelectrodes to monitor electroactive neurochemicals, such as dopamine, epinephrine, norepinephrine, and 5-hydroxytryptophan, enhancing selectivity and temporal resolution.

Despite advancements like waveform alterations enhancing sensitivity in rapid CV [123,124,125], varying chemical basal concentrations pose detection challenges. To achieve sensitivity and selectivity in in vivo electrochemical sensors, the modulation of specific electrochemical processes at the electrode/brain interface is essential. Nanomaterial advancements, encompassing carbon nanotubes, graphene, MOFs (metal–organic frameworks), metals, and metal oxides, bolster electron transfer kinetics. This enhancement elevates sensitivity, diminishes activation overpotential, and minimizes interference from unwanted substances. Additionally, recognition units, such as enzymes, aptamers, and electrochemical probes, play crucial roles in the construction of electroactive neurochemical sensors.

Among these electrode modifiers, metallic nanomaterials exhibit exceptional properties in amplifying electrical signals. Guoyue Shi et al. [126] pioneered the development of a glass-sealed Au nanoelectrode with cluster-like gold nanostructures modified with Nafion, marking the first instance of improving sensitivity and selectivity for the in vivo detection of dopamine (DA). This electrochemical biosensor offers several advantages, including easy insertion, low tissue interference, good biocompatibility, and strong analytical performance. It provides an effective means to monitor brain DA.

In addition to metallic nanomaterials, certain carbon-based nanomaterials possess remarkable electrochemical catalytic properties. Graphene, renowned for its high conductivity, mechanical strength, expansive surface area, and catalytic characteristics [127,128], is extensively used as an electrode in efficient electrochemical sensors. Its wide potential window and rapid electron transfer rate lead to a minimal charge transfer resistance and heightened electrochemical activity. The hydrophobic and p-p interactions between dopamine and graphene enable the distinct separation of the oxidation potentials of dopamine and uric acid. Chen’s group [129] significantly increased the electrode surface area and enhanced the electrocatalytic performance of platinum wires by employing gold nanoparticles (AuNPs) and reduced graphene oxide (rGO)-modified platinum wire microelectrodes on AuNPs/rGO composites, achieving the successful detection of dopamine in the rat brain striatum. Single- and multi-walled carbon nanotubes (SWCNTs and MWCNTs, respectively) were utilized to augment the electroactive/adsorption sites, ensuring a higher sensitivity, and electrocatalytic/defect-enriched sites, ensuring a higher selectivity in monitoring neurochemicals.

In 2019 [130], Taylor et al. enhanced the sensitivity to dopamine by modifying the coating on carbon fiber surfaces, introducing a highly sensitive and selective carbon fiber electrode (CFE). PEDOT finds wide utility in neutral physiological environment analysis for detecting electrophysiological signals and neurotransmission due to its strong adhesion and porous structure. PEDOT/CNT-functionalized sensors have been extensively applied in in vivo DA detection. Cai’s group [131] utilized SWCNTs/PEDOT: PSS-modified microelectrode arrays, creating a four-stalk implantable microelectrode array (MEA) for simultaneously and in real-time detecting bimodal signals—electrophysiological signals and dopamine (DA) concentration—in the rat striatum. This setup effectively monitored the dynamic changes in striatal bimodal signals during isoflurane anesthesia.

To improve the selectivity, some recognition units, such as enzymes, aptamers, and electrochemical probes, have been developed for the detection of electroactive neurochemicals. Mao’s group [132] introduced a novel interface functionalization strategy involving the assembly of aptamer–cholesterol amphiphiles (aptCAs) on alkyl chain-functionalized carbon fiber electrodes (CFEs). This approach proved more effective than pre-treating the electrode surface with a positively charged coating, enabling the efficient immobilization of aptamers on the CFE surface via electrostatic interaction. The resulting modified electrode exhibited increased stability in physiological fluids and demonstrated versatility across various oligonucleotide sequences. Upon implantation into the nucleus ambiguus (NAc) and medial forebrain bundle (MFB) regions of rats, this modified electrode successfully monitored dopamine (DA) dynamics in the living rat brain using amperometry to track changes in DA levels during electrical stimulation. Mao’s group also proposed an electrochemical coupling strategy, covalently placing a specific catechol on the carbon fiber surface, initiating an initial electrochemical coupling on the CFE. This process resulted in a thin layer of quinone intermediates that rapidly bound to thiol-containing oligonucleotides at controlled potentials. This innovative strategy not only simplified and enhanced the efficiency of carbon surface modification but also significantly improved sensitivity and stability in dopamine sensing. It established a robust system for continuously detecting dopamine dynamics in the living animal brain [133].

Based on the reaction of electroactive DBPs with hydrogen peroxide to generate electroactive phenols, a series of phenyl borate derivatives (DBPs) were designed as electrochemical probe molecules. Through analyzing the performance of the synthesized probes, we found that the o-Cl-DBPs exhibited optimal performance. Ratiometric electrodes were successfully constructed using graphene oxide as the internal reference for detection in the three brain regions of Parkinson’s rats. These ratiometric electrodes better mitigated errors arising from differences between in vitro and in vivo environments. Notably, due to graphene’s favorable electrocatalytic effect on ascorbic acid, we unexpectedly discovered the regulation of the balance between the antioxidant AA and hydrogen peroxide in the brain [134].

Researchers have demonstrated a strong interest in other electroactive neurochemicals, developing multiple strategies for in vivo in situ real-time monitoring, as summarized in Table 2.

### 3.2. Microdialysis Coupled with Electrochemical Detection

Microdialysis serves as a pivotal analytical technique for sampling in neurochemical analyses. Especially beneficial for in vivo applications, microdialysis minimizes damage to the brain tissue and exhibits extensive applicability in various extracellular fluid analyses, encompassing heart, fat, liver, and brain tissues [148]. Originating in 1966, the technique relies on analyte diffusion across a porous membrane. Bito’s group [18,149] accessed a dog’s cortical layer using a sterile dialysis capsule, allowing the collection of analytes into a brine stream, subsequently detectable in the flowing salt species. Notably, microdialysis often integrates with chromatography or electrophoresis systems for sample separation. When combined with electrochemical detection systems, it forms a separation-based sensor capable of achieving the highly selective, near real-time monitoring of multiple analytes. However, microdialysis monitoring relying on separation systems requires sample pretreatment, risking sample degradation, causing delays, and limiting spatial resolution due to separation properties. The coupling of microdialysis with biosensors, driven by advancements in electrochemical biosensors, enables the direct analysis of biological samples from living bodies. While less selective than separation-based microdialysis monitoring, this approach’s simplicity negates the need for additional pretreatment, avoids potential analyte degradation, and allows the monitoring of biological events over extended periods, such as 2 h [150].

#### 3.2.1. Detection Techniques with Separation Means

Microdialysis can be paired with separation devices, like high-performance liquid chromatography (HPLC) and capillary electrophoresis (CE), along with associated detectors, to detect samples in dialysate. Continuous efforts have substantially improved microdialysis coupled with separation systems, achieving temporal resolutions in seconds, overturning the prior notion of a poor temporal resolution. As a result, it has also been favored by researchers, with a variety of microdialysis-coupled separation systems emerging as detection strategies that can be applied to the detection of different neurochemicals (Table 3).

**● Microdialysis–capillary electrophoresis/microchip electrophoresis (MD–CE/ME):** MD–CE/ME encounters a challenge due to the high-volume injection requirements (1–10 µL) of conventional separation techniques, leading to a compromised time resolution—a consistent limitation in microdialysis systems. However, the emergence of capillary electrophoresis has significantly alleviated this issue with its high separation efficiency and shorter separation times [151,152,153,154,155]. As early as the 1990s [156,157], the coupling of MD with CE enabled the efficient analysis of polar and charged compounds in biological samples. Initially, the integration of MD and CE was often paired with laser-induced fluorescence (LIF) detection, facilitating the in vivo detection of various neurochemicals, like norepinephrine, glutamate, and aspartate [155,158].

**Figure 5:**
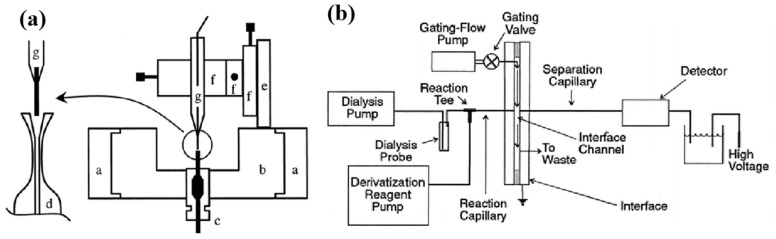
(**a**) Schematic of the etched end-column decoupler and the rotatable detection cell for CE-EC. (a, stationary support collar; b, rotatable detection cell; c, butt connector; d, capillary; e, micropositioner mount; f, three translation stages of the micropositioner; g, carbon fiber microelectrode). Reprinted from Ref. [21] with permission. Copyright 1999 American Chemical Society. (**b**) Block diagram of the microdialysis/capillary zone electrophoresis-LIF system with online derivatization. Reprinted from Ref. [170] with permission. Copyright 1996 American Chemical Society.

Despite CE-LIF detection enhancing the potential for the high temporal resolution monitoring of neurochemicals in microdialysis fluids, it necessitates derivatization to introduce a fluorophore into compounds, limiting its applicability to all metabolites and reducing selectivity for biomolecules. However, capillary electrophoresis (CE) combined with electrochemical detection has emerged as a robust analytical tool [159,160,161]. Thomas J. O’Shea et al. [162] demonstrated the continuous monitoring of amino acids in the brain by integrating CE with electrochemical detection using microdialysis sampling. This setup was showcased by observing “K^+^-induced stimulation of excitatory amino acid release.” Qian’s group [21] developed an integrated end column decoupler with conductive discs in a fused silica capillary wall, providing a low detection limit. This CE-EC system effectively determined dopamine in 1 min brain microdialysis fluid samples from anesthetized rats (Figure 5a).

On the other hand, microchip electrophoresis (ME) not only facilitates rapid MD-LC separation and demands minimal sample volumes but also enables the continuous, simultaneous monitoring of multiple analytes using online separation sensors. When combined with continuous microdialysis sampling, microchip electrophoretic separation-based sensors provide near real-time dynamic information on sample chemical composition. EC detection in ME was first described in 1998 [163] and offers unique advantages [164] over the initially used laser-induced fluorescence techniques [165,166,167]. It allows the direct detection of small, naturally electrically active biomolecules using an amperometric method. Additionally, the integration of microchip electrophoresis separation with the electrochemical system eliminates the need for bulky optical detection instruments, enhancing convenience.

Fabricating MD-ME-EC systems faces challenges in integrating working electrodes into the device. Rachel A. Saylor et al. [168] employed a PDMS/glass hybrid device fabrication process, enabling the successful integration of a high ionic-strength, pressure-driven MD stream with ME and CE on carbon electrodes. This integration allowed the near real-time in vivo monitoring of catecholamines in the rat brain. To enhance electrode alignment reproducibility with the separation channel, Shamal M. Gunawardhana et al. [169] utilized a thermally cracked photoresist film working electrode and a poly (Dimethylsiloxane) microchip with a flow-gated sample injection interface. This setup coupled MD for the online monitoring of levodopa conversion to dopamine and monitoring dopamine release in anesthetized rats after high K^+^ stimulation.

Flow-gated capillary electrophoresis combines conventional CE and microchip CE, using a quartz capillary as a separation channel and adopting a fast flow-gated injection technique primarily from microchip CE. This method utilizes silica capillaries as separation tubes while employing a single-cross microchip configuration for rapid flow-gated injection [20]. As early as 1996 [170], Kennedy’s group investigated capillary electrophoresis coupled with microdialysis using a flow-gated interface for the in vivo quantitative monitoring of compounds like aspartic acid and glutamate in the rat caudate nucleus (Figure 5b). This strategy led to an enhanced separation efficiency and rapid sampling despite trace sample amounts. Subsequently, to further enhance flow-gated CE in vivo monitoring, Bowser and Kennedy [171] reported an improved separation efficiency using smaller diameter capillaries (10 µm), higher electric field strength (2000 V/cm), and sheath-flow cuvettes. Additionally, Kennedy and Bowser [172] utilized derivatization with NDA and NBD-F to enhance sensitivity in detecting primary amines for rat brain neurotransmitter detection.

**● Microdialysis–liquid chromatography (MD–LC)**: MD–LC is a prevalent method for analyzing microdialysis samples, often employing liquid chromatography (LC) coupled with electrochemical, fluorescence, mass spectrometry (MS), or absorbance detection. While capillary electrophoresis boasts a high temporal resolution, HPLC has emerged as the preferred method for studying low concentrations of neurochemicals, like dopamine and 5-HT, due to its reliability and reproducibility. HPLC can provide both base concentrations and dynamic changes. However, conventional liquid chromatography necessitates sufficient dialysate, leading to longer processing times, inadequate temporal resolution, and significant delays between the sample collection and clinical response. Conversely, nano (capillary or microporous)-based LC methods coupled with fluorescence or electrochemical detection offer the selective analysis of neurochemicals in microvolume samples. Previous efforts to enhance the speed of HPLC techniques include using high pressure to increase the linear velocity of the mobile phase, elevating column temperature, and utilizing shorter columns filled with shorter particles [173,174,175]. The temporal resolution of measurements at HPLC not only depends on the sample stream flow rate but also on the sample volume. Capillary columns possess significantly smaller peak volumes compared to the standard or microbore (1 mm diameter) HPLC columns, minimizing the sample volume dilution and greatly improving the temporal resolution. Weber’s group integrated these modifications to determine serotonin (5-HT) levels in the central nervous system of awake animals using capillary LC and electrochemical detection [176]. To further expedite 5-HT chromatographic determinations and enhance the temporal resolution, Weber’s group explored on-column pre-concentration as part of optimization, highlighting the column diameter’s importance in achieving high speed and optimal sensitivity conditions. Smaller particle diameters and higher temperatures were found to yield faster optimal separations and a greater sensitivity. Under ideal conditions, the separation time for 5-HT was reduced to approximately 22.7 s. Online analysis feasibility was demonstrated by simulating repeated injections to achieve a time resolution of 36 s [22].

**Figure 6:**
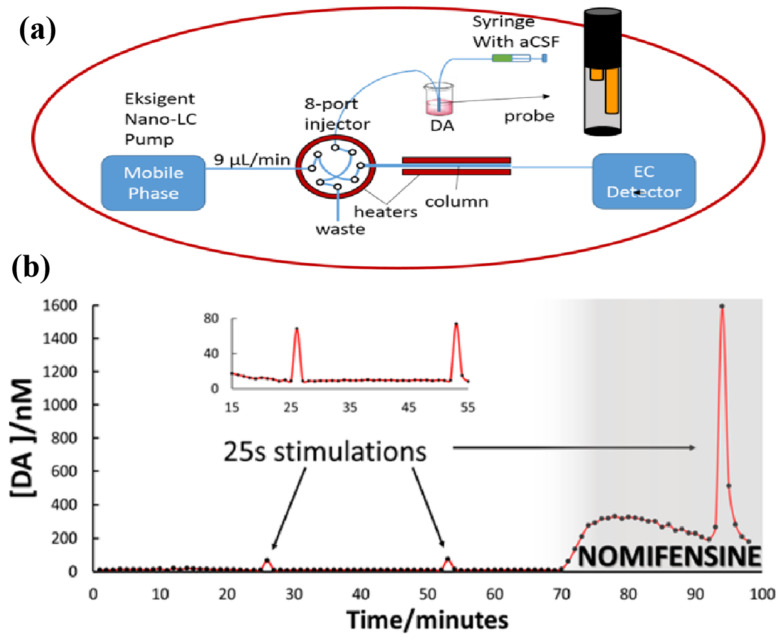
Online capillary liquid chromatography with electrochemical detection. (**a**) In vitro online microdialysis-HPLC-EC experimental system. (**b**) Online in vivo DA measurements. Electrical stimulations were carried out prior to the administration of nomifensine. (The black dots represent the data from the test, and the red lines represent the overall trend). Reprinted from Ref. [178] with permission. Copyright 2015 American Chemical Society.

In another work, the online monitoring of 5-HT in vivo was accomplished by Andrews’ group, employing this rapid microdialysis method in awake animals over extended periods, achieving a temporal resolution of 3 min [177]. This represented a significant advancement. Weber’s group [178] optimized the pH, buffer composition, and surfactant concentration to eliminate interference with dopamine peaks. Employing the system to monitor electrically evoked DA transients, we achieved a true temporal resolution of less than 1 min. The evoked transients were observable in single, 1 min dialysate samples (Figure 6). To ensure robust in vivo online measurements, Weber’s group introduced a six-port recirculating syringe between the syringe pump and the microdialysis probe, utilizing capillary tubing (75 μm inner diameter, 70 cm long) at the inlet and outlet of the probe to minimize solute dispersion. Sampling and analysis were optimized to monitor basal 5-HT concentrations in the striatum of freely moving rats and subsequent responses to 1 min time-resolved stimuli [19]. In another work, Mao Lanqun’s group examined the spontaneous firing rate (SFR) and neurochemical kinetics of the auditory cortex in rats following sodium salicylate injection. They utilized in vivo microdialysis in combination with an online electrochemical system (OECS) and HPLC, revealing significantly elevated levels of glutamate and ascorbic acid [179].

**Table 3:** In vivo detection of neurochemicals in the brain tissue by microdialysis coupled with electrochemical sensing based on separation systems.

Neurochemical	Technique	Sampling Site	Temporal Resolution	LOD	Ref.
DA	MD-LC	Striatum	1 min	-	[180]
DA	MD-HPLC	Nucleus accumbens septi	1 min	-	[181]
DA	MD-HPLC	Striatum	2 min	-	[182,183]
DA	MD-HPLC	Striatum, Cortex	2 min	-	[184]
DA	MD-HPLC	Striatum	1 min	40 nM	[185]
	MD-CapUHPLC	Striatum	1 min	0.15 nM	[178]
DA	MD-ME	Striatum	65	200 μM	[168]
DA	MD-ME	Striatum	100 s	1 μM	[169]
GABA	MD-HPLC	-	1 min	-	[186]
Glu	MD-CE	Striatum	25 s	-	[187]
Glu	MD-HPLC	Hippocampus	1 min	-	[186]
Glu and AA	MD-HPLC	Auditory cortex		0.1/1 μM	[179]
Neuroactive amines and AAs	MD-CapLC	Striatum	10 s	0.09−0.35 nM	[188]
5-HIAA	MD-CapLC	Hippocampus	2 min	3 nM	[189]
5-HT	MD-CapLC	Hippocampus	1 min	56 μM	[189]
5-HT	MD-HPLC	Hippocampus	90 s	-	[186]
5-HT	MD-HPLC	Striatum, Cortex	2 min	-	[184]
5-HT	MD-HPLC	Hippocampus, Striatum	3 min	0.8 fM	[177]
5-HT	MD-CapUHPLC	Hippocampus	1 min	70 pM	[176]
5-HT	MD-CapUHPLC	Striatum	36 s	0.3 nM	[22]
5-HT	MD-CapUHPLC	Striatum	30 s	160 pM	[19]
NA, DA, and 5-HT	MD-UHPLC	Prefrontal cortex	12 min	32/42/83 pM	[190]
NA, DA, and 5-HT	MD-CapUHPLC	Striatum, Amygdala, Hippocampus	21 min	0.75/0.75/1.5 nM	[191]
NA, DA, and 5-HT	MD-UHPLC	Hippocampus	8 min	83/58/60 pM	[192]
Monoamines	MD-UHPLC	Hippocampus, Prefrontal cortex, Striatum	20 min	100 pM	[193]
Ach	MD-HPLC	Striatum	7 min	20 fmol	[194]
Ach	MD-HPLC	Hippocampus	6 min	10 fmol	[195]

LC: liquid chromatography; HPLC: high-performance liquid chromatography; CapUHPLC: capillary ultra-high-performance liquid chromatography; ME: microchip electrophoresis; CE: capillary electrophoresis; CapLC: capillary liquid chromatography; UHPLC: ultra-high-performance liquid chromatography.

#### 3.2.2. Detection Techniques with Biosensors

The use of biosensors offers a viable alternative to separation devices, which often necessitate bulky hardware and intricate operational mechanisms. Coupling biosensors with microdialysis introduces near real-time capabilities and shorter analysis times. This method involves fewer technical requirements as it avoids the need for sample collection and separation. While the use of biosensors has significantly improved the drawback of low temporal resolution, other challenges have emerged, including issues related to stability, reproducibility, etc. [196]

An ideal biosensor should have the capability to continuously and reliably monitor analytes in complex bodily fluids from living organisms over an extended period. Typically, a biosensor comprises one or more recognition elements designed to identify target molecules [197,198]. This process generates a chemical signal captured by the sensor component and translated into an optical or electrochemical signal (Table 4).

The utilization of enzymes stands as a common strategy in biosensors. In 2005, Mao’s group [25] summarized the principles, development, and notable applications of early enzyme-based continuous online monitoring combined with microdialysis sampling and biosensors. Notably, their work involved the development of enzyme-based biosensors integrated with microdialysis, employing thionine and xanthine oxidase (XOD) as low-potential mediators and oxidases [23]. This demonstrated that the use of low-potential mediators for electron transfer in oxidases offers a novel approach for developing oxidase-based biosensors, both theoretically and technologically simple.

**Table 4:** In vivo detection of neurochemicals in the brain tissue using microdialysis coupled with electrochemical biosensors.

Neurochemical	Technique	Sampling Site	Temporal Resolution	LOD	Ref.
DA	MD-Biosensors	Striatum	4 min	0.31 nM	[199]
DA	MD-Biosensors	Striatum	10 min	-	[200]
Glucose and lactate	MD-Biosensors	Cortex	30 s	-	[201,202,203]
Glucose and lactate	MD-Biosensors	Cortex	1 min	-	[204]
Glucose and lactate	MD-Biosensors	Striatum	-	2.39/2.52 µM	[205]
Lactate	MD-Biosensors	Striatum	1 min	-	[206]
Glucose	MD-Biosensors	Cortex	2 min	50 nM	[207]
Glucose	MD-Biosensors	Auditory cortex	-	10 µM	[208]
Glucose	MD-Biosensors	Striatum	-	1.8 µM	[209]
Glucose	MD-Biosensors	Striatum	-	0.28 µM	[210]
Glucose	MD-Biosensors	Striatum	-	3.33 µM	[211]
Ach	MD-Biosensors	Striatum	-	1 µM	[24]
Hypoxanthine	MD-Biosensors	Striatum	-	0.40 µM	[23]
Cu^2+^	MD-Biosensors	Striatum	-	13 nM	[212]
ATP	MD-Biosensors	Cortex	-	50 pM	[213]
ATP	MD-Biosensors	Cortex	0.5 s	0.1 fmol	[214]
H_2_O_2_	MD-Biosensors	Cortex, Striatum, Hippocampus	-	1 µM	[79]

DA: dopamin; Glu: glutamate; Ach: acetylcholine; ATP: adenosine triphosphate; MD: microdialysis.

The combination of microdialysis enables the highly selective detection of endogenous species within the brain system. Their research showcased an online electrochemical system (OECS) for the selective and continuous measurement of acetylcholine (ACh) [24], achieved by efficiently integrating in vivo microdialysis, a multienzymatic microreactor, and an electrochemical detector.

**Figure 7:**
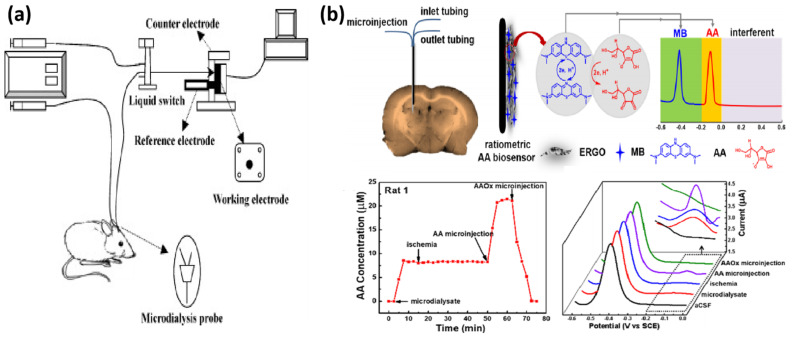
Several microdialysis-based sampling combined with electrochemical biosensors for the in vivo analysis of neurotransmitters. (**a**) Schematic presentation of the online microdialysis system with GlutaOx-modified biosensors for the continuous monitoring of glutamate. Reprinted from Ref. [215] with permission. Copyright 2023 Elsevier B.V. (**b**) Construction of a RECS for the selective determination of AA in cerebral microdialysis fluids and changes in AA concentration under whole-brain ischemia–reperfusion. Reprinted from Ref. [217] with permission. Copyright 2020 American Chemical Society.

In a separate study, Yanyan Yu et al. [215] synthesized a new room-temperature ionic liquid, [C_3_(OH)_2_mim][BF_4_], with two hydroxyl functionalities in the imidazolium core. This introduction of functional groups significantly enhanced the stabilization of Au/Pt alloys and facilitated the formation of well-dispersed small metal nanoparticles, exhibiting a good catalytic activity against hydrogen peroxide. Using glutamate oxidase as a biorecognition element, the continuous detection of glutamate was achieved in the rat striatum, observing normal levels and changes in concentration following various stimuli (Figure 7a).

To further enhance in vivo sensing accuracy and selectivity, a dual recognition unit strategy (DRUS) was proposed. Yanyan Yu et al. [214] constructed a DRUS ATP biosensor with high selectivity and sensitivity. Utilizing aptamer-to-base and polyimidazole-to-phosphate recognition abilities, the biosensor exhibited an ultra-high sensitivity to subinosinazole levels of ATP-LOD and a remarkable selectivity against interfering ADP and AMP sensing in the extracellular ATP microdialysate sampled from the brain system.

Ratiometric electrochemical sensors (RECSs) have shown improved reproducibility, stability, and reliability in correcting errors during in vivo sensing, making them ideally suited for repetitive in vivo analyses [9,212,216]. Building upon this concept, Gu’s group constructed RECSs by modifying graphene oxide on electrodes via electrodeposition. Subsequently, methylene blue was adsorbed onto the graphene oxide using electrostatic attraction, serving as an effective internal reference ratio. The introduction of graphene sp3 C-C defects via subsequent electroreduction facilitated the electrochemical oxidation of AA at low potentials, ensuring a high selectivity in the brain system against potential interferences. One point that is worth noting is that this voltammetric RECS to accomplish in vivo/online repetitive measurements included a time interval of more than 1 min, which is capable of tracking the physiological process happening over several minutes [217] (Figure 7b).

All in all, the integration of microdialysis with electrochemical systems offers advantages, such as simple equipment, low cost, and high sensitivity. Initially, the coupling of microdialysis with separation systems and integrated electrochemical detection was primarily used for detecting electroactive neurochemicals. As the coupling extended to biosensors, designing recognition units on the sensor surface allowed the detection of not only electroactive neurological substances but also non-electrochemically active neurochemicals. However, the sometimes slow electrochemical reaction rates and coupled biocatalytic processes limit the temporal resolution. Spectroscopic methods, such as laser-induced fluorescence (LIF) and visible light absorption, have fast reaction kinetics. The application of microdialysis in combination with spectroscopic techniques has been shown to provide a high temporal resolution for the detection of neurochemicals. Additionally, integrating microdialysis with mass spectrometry serves as a powerful tool for analyzing biologically active molecules. This integration provides sequence specificity and enhanced mass sensitivity, enabling detection across a broad range of analytes. These assays significantly compensate for the limitations of integrated microdialysis and electrochemical detection platforms. As this review primarily focused on the application of electrochemical sensing to the brain tissue, we thus omitting detailed descriptions of other assays.

### 3.3. Emerging Techniques

**Figure 8:**
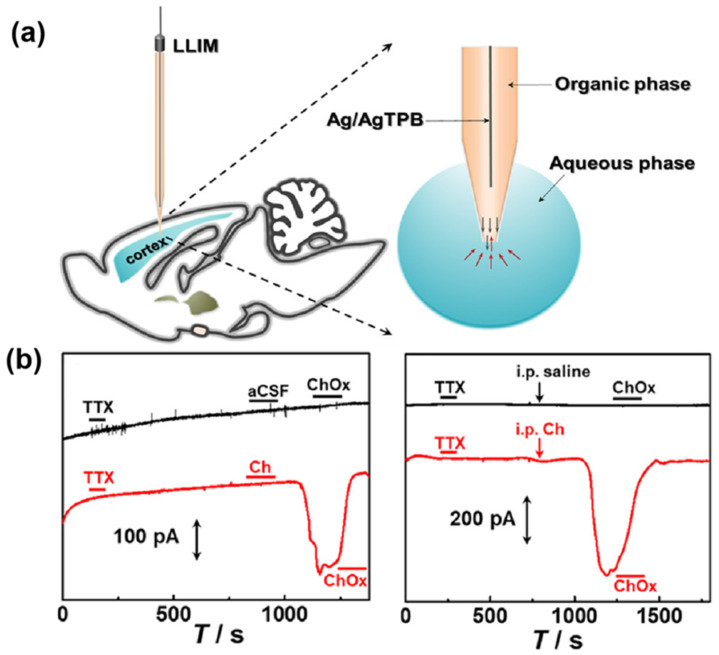
(**a**) Illustration of a measurement using the LLIM in the rat brain. (**b**) Typical amperometric responses obtained using the LLIM in the cortex of anesthetized rats. Reprinted from Ref. [218] with permission. Copyright 2021 American Chemical Society.

In recent years, a liquid/liquid interface microsensor (LLIM) using a nano-micropipette emerged, utilizing electrochemical techniques to monitor the charge transfer from the aqueous phase to the organic phase of an analyte. This innovation allows the monitoring of neurochemicals in the living brain, even those lacking redox activity. Electrochemistry at the liquid/liquid interface, or the interface between two immiscible electrolyte solutions, has advanced the direct analysis of ions by leveraging the difference in electrolytic energy between the ionic solvents of neighboring phases. Adjusting the pipette tip to the micrometer scale enhanced the spatial resolution of in vivo analyses. In recent decades, electrochemical sensing at liquid/liquid interfaces has garnered attention for analyzing non-electrochemically active substances, like neurochemicals, amino acids, peptides, and proteins. For example, choline (Ch) exhibits specific ion transfer potential and a distinct ion transfer current signal. Zhang’s group [218] employed 1,2-dichloroethane as the organic phase and choline-containing rat cerebrospinal fluid as the aqueous phase, utilizing the disparity in solvation energies between the liquid phases. This approach demonstrated a good linearity and selectivity in responding to Ch, achieving a detection limit of 0.37 μM (Figure 8). Also, nanotechnology and liquid–liquid interfacial sensing have combined to enable neuronal monitoring for neurochemicals. Novel sensing principles based on nanochannels, particularly ionic current rectification (ICR) technology, have been developed. Lanqun’s group explored ICR at the micrometer scale [219], unveiling a new strategy using the microscale to selectively sense ATP in the brain system. They employed polyimidazole [220] to modify the inner wall surface of the microtubule, offering a good linearity for ATP within the 5–100 nM concentration range through differential binding between the positively charged polyimidazole and negatively charged ATP aptamers.

In another study, field-effect transistor (FET) technology, highly sensitive and selective in biosensing, allows miniaturization and has gained prominence for real-time, high-throughput, and high-sensitivity assays. Among biosensing platforms, FET biosensors excel in enzyme-modified biosensors due to their precise analyte detection, small size, integrated compactness, and potentially cost-effective quality. These sensors detect hydrogen ions (H^+^), byproducts produced by enzymes and analytes, proportionate to the analyte concentration. Graphene, with its unique structural geometry, excellent electrical properties, biocompatibility, and sensitivity to surface charge alterations, serves as an active channel for immobilizing enzyme molecules in FET biosensors. Hwang et al. [221] developed a reduced graphene oxide-based enzyme-modified FET (RGO-EnFET) to study acetylcholinesterase enzyme kinetics and the impact of acetylcholinesterase inhibitors on AD therapy. Additionally, Fenoy’s group [222] proposed modifying the FET graphene channel with a copolymer poly(3-aminobenzylaminobenzidine-co-phenylene amine) (PABA) film, enhancing the electrostatic charge and creating a non-denaturing environment for enzyme immobilization. This improved the FETs’ pH sensitivity, enabling real-time detection of acetylcholinesterase within the 5–1000 μM range for acetylcholine sensing.

FET technology has also integrated aptamers. Andrews’ group [223] obtained DA- and 5-HT-specific aptamers through SELEX screening, constructing flexible aptamer field-effect transistor sensors for DA and 5-HT detection. These sensors successfully detected target analytes in artificial cerebrospinal fluid and mouse brain slices.

The quality of behavioral correlates of neurochemical measurements can be improved as new wireless data transmission systems are able to perform the tests without tethering the animal [224]. As physics, neurophysiology, chemistry, and other disciplines continue to advance, microelectronic devices for wireless neurochemical sensing have been developed to detect neurochemicals in non-tethered animals. In addition, wireless transmission systems can also be used in human medicine. Roham et al. [225] proposed an integrated chip for wireless neurochemical measurements that provides both amperometry and fast scanning cyclic voltammetry. With the continued efforts of researchers, wireless transmission system test instruments are slowly becoming portable and wearable. Tonello et al. [226] have developed a low-cost, high-sensitivity, portable point-of-care (PoC) detection system based on a screen-printed electrochemical sensor that used a proprietary antibody to detect unfolded p53, enabling the detection of this biomarker in Alzheimer’s patients.

In recent years, Drakakis’ group [227] has developed a battery-powered potentiostat and wireless data transmission system that includes integrated biosensors and a microfluidic system for microdialysis. It can detect glucose and lactate in the brain of brain-injured patients using amperometry and potassium ions using potentiometry.

## 4. In Vitro Electrochemical Measurements of Biomolecules in the Brain Tissue

Both the in vivo and in vitro assays of neurochemicals play crucial roles in exploring brain science. Conducting detections on brain slices in vitro helps to avoid interference from the in vivo environment. Isolated brain slice cultures serve as common analytical models for studying neurophysiology, frequently employed for monitoring neurochemicals in the brain tissue in vitro. Moreover, multiple slices from the same brain can be obtained and measured, allowing access to deeper tissue regions. The experimental setup is typically more convenient with brain slices. However, brain-sectioning experiments come with limitations. The tissues undergo mechanical trauma during sectioning, raising concerns about whether measurements are taken from healthy or injured regions. Moreover, phenomena like Donnan swelling, due to the exposure of intracellular charged molecules during sectioning, are likely to alter the tissue’s mechanical properties.

**Figure 9:**
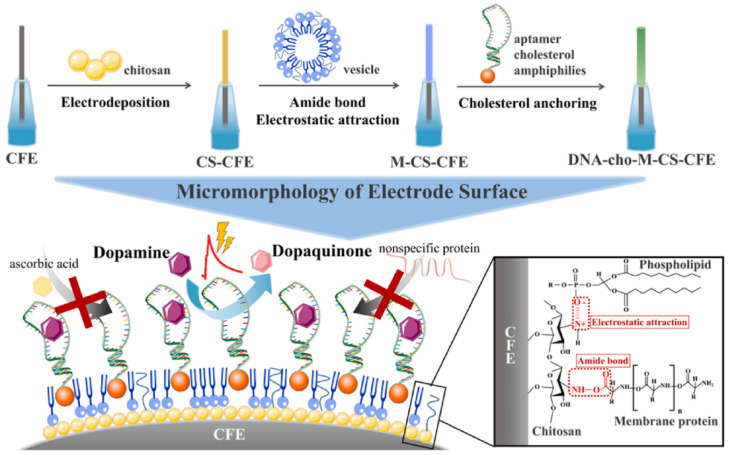
Schematic illustration of the fabrication of DNA-cho-M–CS–CFE and the DA measurement in the micromorphology of the electrode surface. (The red dotted box is the binding sites of chitosan to phospholipids and membrane proteins, respectively.) Reprinted from Ref. [228] with permission. Copyright 2023 Elsevier B.V.

In vivo assays are more time-sensitive compared to in vitro assays, enabling the real-time tracking of dynamic changes in target analyte concentrations throughout the organism. Yet, the complexity of the in vivo environment poses additional detection challenges. Both in vitro and in vivo assays possess strengths and weaknesses, continuously evolving and progressing in parallel, complementing each other with their respective strengths. In this regard, Wu’s group [228] enhanced electrode biocompatibility and stability by modifying the CFE with chitosan (CS) membranes, brain cell membranes, and the aptamer cholesterol amphiphile (DNA-Cho). Their electrode demonstrated a high sensitivity, specificity, and stability in detecting DA and was utilized to detect potassium-ion-induced DA release in brain slices and PC12 cells, unveiling the specific process of DA release inhibition by lipopolysaccharide (Figure 9). This robust electrode modification strategy facilitates the in vivo monitoring of DA sensing activities in complex environments. The development of microarray probes (MEAs) has offered new directions for researchers. Hossain et al. [229] designed and validated a platinum (Pt) microelectrode array-based GABA probe, utilized for in vitro measurements in brain slices. The probe includes two microbial sensors, with the concentration of GABA corrected by the difference in oxidation currents of hydrogen peroxide generated during measurements by the two microbial sensors. This approach minimizes the impact of the complex brain tissue environment. To enhance enzyme stability, Asri’s group [230] developed an enzyme-coating method that significantly optimized the mechanical stability, enabling its use in in vitro slices for selective acetylcholine detection. However, compared to in vivo monitoring, in vitro assays do not fully reflect the overall physiological trends in organisms, leading researchers to prefer in situ real-time monitoring in vivo.

## 5. Conclusions and Outlook

The accurate measurement of fast neurochemicals within the central nervous system remains pivotal in neuroscience, offering a comprehensive understanding of the molecular mechanisms driving physiological and pathological brain processes. Electrochemical sensing stands as a potent tool for monitoring small biomolecules in the brain tissue. Implantable electrochemical sensors and microdialysis represent two complementary categories in electrochemical sensing. Implantable sensors, providing high temporal and spatial resolution, enable the real-time monitoring of neurochemical signals in specific brain regions. Their sub-second temporal resolution allows for the study of rapid events associated with specific neurophysiological activities, eliminating isolation processes.

Nanomaterial-based electrochemical sensors, like graphene, carbon nanotubes, molecularly imprinted polymers, metal–organic frameworks, and metal nanoparticles, showcase robustness, selectivity, sensitivity, precision, and accuracy in quantifying neurochemicals. These sensors excel in measuring target molecules amidst complex physiological environments. Further advancements in recognition units (e.g., enzymes, aptamers, and molecularly imprinted polymers) have enhanced selectivity, improving the reliability of in vivo measurements. However, challenges, such as foreign body reaction from implantable electrodes, electrode contamination, and protein adsorption, still impede the stability and accuracy of long-term monitoring.

The development of flexible and degradable materials holds promise for sustained long-term bioanalytical tracking. Microdialysis, a potent sampling technique, facilitates the continuous monitoring of biomolecule concentrations in vivo and in vitro. Its broad sampling range encompasses diverse small molecules, like neurochemicals, amino acids, and neuropeptides, enabling simultaneous detection in microdialysates.

Though microdialysis suffers from a poor spatial and temporal resolution, its coupling with separation techniques, like high-performance liquid chromatography and capillary electrophoresis, has alleviated this drawback. Integrating microdialysis with electrochemical biosensors circumvents the need for sample separation. However, biosensors often rely on specific enzymes or aptamers, limiting target molecule detection. Moreover, costly microdialysis probes and the susceptibility of dialysis membranes to clogging pose challenges for reuse.

Continuous advancements in microdialysis probes, refining capillary column diameters, and optimizing biosensor surface construction strategies have propelled microdialysis in neurochemistry. The current neurochemical detection focuses on in vivo measurements in freely moving or conscious mice/rats, aligning studies closer to natural conditions. Emerging technologies, like ionic current rectification and field-effect transistors, open new frontiers and wireless data transmission systems for in vivo electrochemical sensing. Future research directions may focus more on the real-time in situ measurement, visualization, and simultaneous detection of multiple neurochemicals without interference. Advanced clinical neurotransmitter measurements are anticipated to develop, enriching neuroscience research, with electrochemical neurotransmission analysis poised as a potent tool in disease diagnosis and treatment.

## Figures and Tables

**Table 1:** Implantable electrochemical biosensors to monitor non-electroactive neurochemicals in the brain tissue in vivo.

Neurochemicals	Sensor Structure	Linear Range	LOD (μM)	Detection Area	Ref.
ATP	Au/Apt/3-MPA	0.001–100 µM	0.5	Cortex	[73]
Cl^−^	CFME/OxGO/Ti_3_C_2_T_x_/Ag	1–700 mM	10	Hippocampus	[74]
Cl^−^	CFME/GO/TNWs/Ag/MB	1–300 mM	10	Hippocampus	[75]
H^+^	CFME/EOGO/PMe	0.5–600 μM	0.036	Hippocampus	[76]
H^+^	CFNE/CNTs/PoPD	4.5–8.2 pH	-	Hippocampus	[77]
H^+^	Hemin-Fc/CNF	5.5–8.0 pH	-	Striatum, Cortex	[78]
H^+^	Cat + Fc/SWNT/CFME	5.91–7.81 pH	-	Striatum	[79]
K^+^	NO/K^+^ dual microsensor	0.01–100 mM	-	Cortex	[80]
H^+^, K^+^, Ca^2+^, and Na^+^	Mesoporous SiO_2_/carbon/Co(II) phthalocyanine	0.1–70.79 μM	-	Hippocampus	[81]
		50 μM–140 mM			
		1 μM–160 mM			
		130 μM–200 mM			
O_2_^•−^	CFME/SWCNT/MB + ND	2–200 μM	0.52	Striatum, Cortex, Hippocampus	[82]
Glucose	Gox/PB/PANI/MWNT/CFE	50−4000 μM	40	Cortex	[83]
Lactate	Pt-ceria biosensors	100 pM−15.5 mM	0.1	Hippocampus	[84]
K^+^	NO/K^+^ dual microsensor	10 μM−100 mM	-	Cortex	[80]
Glutamate	Pt-Ir/PPD/GlutOx/AsOx/BSA	5–150 µM	0.044	Subthalamic nucleus	[11]
Glutamate	GluOx/pDAB/polyimide	Up to 150 µM	0.22	Cortex	[85]

ATP: adenosine triphosphate; Apt: aptamer; 3-MPA: 3-Mercaptopropionic acid; CFME: carbon fiber microelectrode; OxGO: electro-oxidized graphene oxide; Ti_3_C_2_T_x_: two-dimensional MXene titanium carbide; GO: graphene oxide.; TNWs: titanate nanowires; MB: methylene blue; EOGO: electrochemically oxidized graphene oxide; PMe: poly-(melamine); CFNE: carbon fiber nanotip electrode; CNTs: carbon nanotubes; PoPD: poly-o-phenylenediamine; Hemin-Fc: hemin-aminoferrocene; CNF: carbon nanotube fiber; Cat: catalase; Fc: ferrocene; SWNT: single-walled carbon nanotube; ND: diphenylphosphonate-2-naphthol ester; Gox: glucoseoxidase; PB: Prussian blue; PANI: polyaniline; MWNTs: multi-walled carbon nanotubes; CFE: carbon fiber electrode; PPD: poly-o-phenylenediamine; GlutOx: glutamate oxidase; AsOx: ascorbate oxidase; BSA: bovine serum albumin; pDAB: poly(1,3-diaminobenzene).

**Table 2:** Implantable microsensors for the detection of electrochemically active neurochemicals in the brain tissue in vivo.

Neurochemicals	Sensor Structure	Linear Range	LOD	Detection Area	Ref.
DA	PTA-PANI-coated/CFE	5–30 µM	-	Striatum	[16]
DA	PB/PEDOT/CF_disk_	0.5–10 mM	0.18 µM	Striatum	[135]
DA	PEDOT/GO/CFE	6.25–212.5 µM	-	Dorsal Striatum	[136]
DA	Nafion-Au/GCNE	0.02–5.6 µM	0.01 µM	Striatum	[137]
DA	AuNPs-rGO/Pt	0.05–3 µM	0.01675 µM	Striatum	[129]
5-HT	CFMEA/DS-SWCNT	0.10–3.40 µM	5.1 µM	Striatum	[10]
5-HT	CFEA/GR-FeTSPc	0.05–60 µM	0.02 µM	Hippocampus	[138]
5-HT	PEDOT/CNT-coated	0.01–1 µM	-	Hippocampus	[139]
AA	SWCNT/CFE	10–1000 µM	1000 µM	Cortex	[140]
AA	PEDOT/EOGO/CFE	20–1000 µM	500 µM	Striatum, Cortex, Hippocampus	[141]
Cu^2+^	CFME/SWNT + AQ + NS4-C_1_ + ABTS	0.5–9.5 µM	500 nM	Striatum, Cortex, Hippocampus	[142]
Cu^2+^	CFME/Au/E_2_Zn_2_SOD/Ni-NTA	0.01–35 µM	3 nM	Striatum	[143]
H_2_O_2_	PDA/PB/CNT/CFE	0−2775 μM	0.12 μM	Cortex	[144]
H_2_O_2_	Cat/ Nafion-PPD/Pt	25−1000 μM	1.0 μM	Striatum	[145]
H_2_O_2_	Cat + Fc/SWNT/CFME	1.0–230 mM	-	Striatum	[79]
H_2_S	CFE/mAu/M_PS-1_ + M_HS-1_	0.2–40 μM	47 ± 4 μM	Cortex, Striatum, Hippocampus	[9]
H_2_S_n_	CFE/Au/FP2 + FcBT	0.25–20 μM	50 μM	Cortex, Striatum, Hippocampus	[146]
NO	NO/K^+^ dual microsensor	0–3.13 μM	-	Cortex	[80]
NO	CFE/Ni-P/17-FTMS	1–3 μM	12.1 ± 3.4 nM	Cortex	[147]

PTA: polytannic acid; PANI: polyaniline; CFE: carbon fiber electrode; PB: Prussian blue; PEDOT: poly(3,4-ethylenedioxythiophene); CF_disk_: carbon fiber disk; GCNE: glass capillary nanoelectrode; AuNPs: gold nanoparticles; rGO: reduced graphene oxide; CFMEAs: carbon fiber microelectrode arrays; DS: diazonium salt; SWCNTs: single-walled carbon nanotubes; CFEAs: carbon fiber electrode arrays; GR-FeTSPc: graphene-iron-tetrasulfophthalocyanine; AQ: 9,10-anthraquinone; NS4-C_1_: N,N-bis(2-[2-(ethylthio)ethyl])-2-naphthamide; ABTS: 2,2′-azino-bis(3-ethylbenzthiazoline-6-sulfonic acid); E_2_Zn_2_SOD: Cu-free derivative of bovine erythrocyte copper–zinc superoxide dismutase; PDA: polydopamine; PPD: poly-o-phenylenediamine; Cat: catalase; Fc: ferrocene; mAu: mesoporous gold film; M_PS-1_: 3,4-bis((2-fluoro-5-nitrobenzoyl)oxy)-benzoic acid; M_HS-1_: N-(4-(2,5-dinitrophenoxy) phenyl)-5-(1, 2-dithiolan-3-yl)pentanamide (MHS-1); FP2: 4-(5-(1,2-dithiolan-3-yl)pentanamido)-1,2-phenylene bis(2-fluoro-5-nitrobenzoate); FcBT: α-lipoic acid ferrocenylamide; Ni-P: nickel(II)Tetrakis 3-methoxy-4-hydroxyphenyl-porphyrin; 17-FTMS: (heptadecafluoro-1,1,2,2-Tetrahydrodecyl) trimethoxysilane.
